# Outline of a project for nursing health education on the Instagram social network

**DOI:** 10.1590/0034-7167-2022-0301

**Published:** 2023-03-27

**Authors:** Gabriella Picoli dos Santos Faustino, Matheus Oliveira da Silva, Antonio José de Almeida, Márcia de Assunção Ferreira

**Affiliations:** I Universidade Federal do Rio de Janeiro. Rio de Janeiro, Rio de Janeiro, Brazil

**Keywords:** Health Education, Social Networking, Health Promotion, Technology, Nursing., Educación en Salud, Red Social, Promoción de la Salud, Tecnología, Enfermería., Educação em Saúde, Rede Social, Promoção da Saúde, Tecnologia, Enfermagem.

## Abstract

**Objectives:**

to characterize the outline of a project for health education and its contributions to the propagation of information on the Instagram social network.

**Methods:**

exploratory and descriptive research on an Instagram profile called “@resenhadasaude”;. Data collection: from July 23, 2020, to April 21, 2021. Interaction metrics were generated on 36 posts. Simple and percentage statistical analysis were applied.

**Results:**

there are 1,016 followers in Brazil, with a 206.02% growth. The largest audience is teenagers, young people, and women, with a gender difference of 41.8%. The greatest interest was about covid-19, sexual health, and drugs. Followers’ misconceptions reinforce the need for the dissemination of quality information.

**Conclusions:**

Instagram metrics point to the project’s validation in terms of audience interest, mostly adolescents and youth. Instagram proved to be powerful for educational purposes and information dissemination, as well as an autonomous field for nursing.

## RESUMO

**Objetivos:**

caracterizar o perfil de um projeto de educação em saúde e suas contribuições à difusão de informação na rede social Instagram.

**Métodos:**

pesquisa exploratória e descritiva sobre um perfil do Instagram denominado “@resenhadasaude”;. Coleta de dados: de 23 de julho de 2020 a 21 de abril de 2021. Geraram-se métricas de interação sobre 36 postagens. Aplicou-se análise estatística simples e percentual.

**Resultados:**

são 1.016 seguidores no Brasil, com crescimento de 206,02%. Maior público é adolescente, jovem e feminino, com diferença entre os gêneros de 41,8%. O maior interesse foi sobre a covid-19, saúde sexual e drogas. Equívocos de conhecimento dos seguidores reforçam a necessidade de difusão de informações de qualidade.

**Conclusões:**

as métricas do Instagram apontam a validação do projeto em termos de interesse do público, majoritariamente de adolescentes e jovens. O Instagram mostrou-se potente para fins educativos e difusão de informação, bem como campo autônomo para a enfermagem.

## RESUMEN

**Objetivos:**

caracterizar perfil de un proyecto de educación en salud y suyas contribuciones a la difusión de información en la red social Instagram.

**Métodos:**

investigación exploratoria y descriptiva sobre un perfil del Instagram denominado “@resenhadasaude”;. Recogida de datos: de 23 de julio de 2020 a 21 de abril de 2021. Generadas métricas de interacción sobre 36 publicaciones. Aplicado análisis estadístico simple y porcentual.

**Resultados:**

son 1.016 seguidores en Brasil, con crecimiento de 206,02%. Mayor público es adolescente, joven y femenino, con diferencia entre los géneros de 41,8%. Mayor interés fue sobre covid-19, salud sexual y drogas. Equívocos de conocimiento de seguidores refuerzan la necesidad de difusión de informaciones de calidad.

**Conclusiones:**

las métricas del Instagram apuntan la validación del proyecto en términos de interés del público, mayoritariamente de adolescentes y jóvenes. El Instagram se mostró potente para fines educativos y difusión de información, así como campo autónomo para a enfermería.

## INTRODUCTION

The Brazilian internet started to be deployed in Brazil as a communication infrastructure for academic purposes in 1989. In 1994, the network was opened to the general public, the so-called “commercial internet”, with a service distribution outside the academic circles by free private initiative^(1)^. Since then, it has become exponentially popular and is currently used in more than 80% of Brazilian homes. Available in 94% of homes, the cell phone is the most used equipment to access the service^(2)^, indicating a more democratized access to the internet and information, so restricted decades ago.

There are 134 million Brazilian internet users, and most activity is related to communication, led by instant messaging (92% of users), followed by the use of social networks (76%)^(3)^. A study on trends that shape the world, exploring the impact of the Internet, showed that eight out of ten individuals access websites with health-related information. Calculations indicate that the number of Brazilian Internet users regularly accessing these sites may exceed 10 million^(4)^.

There is no doubt, therefore, that the advent of the Internet has allowed broad access to information. Among the means to access it, we find social networks, platforms with high data generation^(5)^. Considering this, searches for topics on diseases and self-care have expanded, which allows for an easier access of this population to professionals. It is also considered that social media enables collaborative learning through interaction with knowledge sharing and involvement of participants^(6)^, allowing articulation among users through comments, forms, and private messages, for example.

Digital, social, and mobile technologies have been widely used in general professional health education and, particularly, in nursing^(7-8)^; in this context, social media have been used on a global scale as a fundamental vehicle for communication. Due to their ease of access, nurses have adopted and actively used them in favor of the population’s health, which has generated a series of successful initiatives led by nurses^(9)^.

Nevertheless, the potential risks that they bring cannot be neglected^(9)^. Thus, considering the importance of social networks and the speed of data dissemination, it is essential to validate this information, because some of it is scientifically erroneous and/or incomplete^(10)^. As such, when people resort to the Internet in search of answers to their health problems, they are susceptible to a mistaken self-diagnosis, which can contribute to an inappropriate self-prescription, generating problems for self-care and worsening of clinical conditions. These misconceptions produce doubts about the information raised in the network^(11)^.

In order to authenticate scientific knowledge accessible to the population, and considering the extreme relevance that social networks have acquired in the modern world, it is necessary to innovate. Social media are configured as a transforming technology in communications in this century, in the field of business, entertainment, and in education^(12)^. The use of social networks as an educational strategy is a challenge, and Instagram is one of these networks that have been used with pedagogical intent, enabling educational studies in the areas of humanities and exact sciences, for example^(13)^. Moreover, it is widely applied in health contexts, such as patient care, monitoring, rehabilitation, diagnosis, teaching, and research^(14)^.

Instagram was launched in 2010 and, since then, has been evolving and improving its tools for dissemination of content through texts, images, and sound, including the possibility of live broadcasts, marketing of products and services, association with anti-bullying tools, positioning itself in favor of the users’ well-being^(15)^. By July 2022, the network had at least 1.440 billion users worldwide^(16)^. In Brazil, by the beginning of 2022, it had 119.5 million users, but it is worth mentioning that the platform restricts access to people aged 13 or older; therefore, the number of Brazilian users is equivalent to 67.4% of the country’s population^(17)^.

This whole apparatus of digital platforms is part of what is called “information and communication technologies” (ICTs), which unite computer technologies with telecommunications technologies; and, in turn, when they acquire educational purposes, they integrate the subdomain of educational technologies^(18)^.

ICT resources, such as social networks, have been increasingly used in the dissemination of scientific and reliable knowledge to the population, making it possible to reach not only health professionals, but to contribute to the democratization of knowledge for society in general^(19-21)^.

Integrative review conducted on the use of ICTs in health education for adolescents included publications from 2014 to 2020 and showed gaps in the production of scientific articles on the subject in Brazil; despite this, it indicates that nursing professionals stand out in the construction of these technologies^(22)^. Thus, it is considered convenient and timely to generate knowledge that gives visibility to initiatives to use social networks to disseminate knowledge to the population, through projects developed by nursing.

## OBJECTIVES

To characterize the outline of a project for health education and its contributions to the propagation of information on the Instagram social network.

## METHODS

### Ethical aspects

This research was conducted based on a database generated by the tools of Instagram itself and did not involve direct invitation of its page followers, so it was not necessary to submit it to the Ethics Committee. The researchers are the administrators of the profile, and all have institutional authorization to manage it according to a term of commitment on maintaining data confidentiality. As such, the data were anonymized, following what is recommended by Art. 7 of Law No. 13,709, of August 14, 2018, which deals with the protection of personal data.

### Study design, period, and data source

Exploratory and descriptive research on an Instagram profile called “*Resenha da Saúde*” [Health Review] (@resenhadasaude), whose purpose is to spread knowledge on health promotion and self-care. Due to the chosen research typology, data were generated to carefully learn more about this profile, through its constitutive characteristics^(23)^.

This profile contains weekly posts, a form created using the Google Forms tool for the public to submit questions, a weekly Quiz published on the Stories feature, created as a tool for testing users on the topic of the week, and short educational videos published using the Reels tool.

The profile is managed by two nursing professors and a team of four to six nursing students from a public federal university located in the city of Rio de Janeiro. Posts are published weekly, with diverse themes, aiming at the adolescent and young public. Each theme is addressed by a student of the team, under the supervision of the teachers.

The data were obtained from the profile itself, whose first post was made on July 23, 2020. As such, the data collection period spanned from the date of the first post to December 11, 2021.

The Instagram social network has a variety of tools and resources^(15)^: it provides metrics to measure several factors of a profile and its publications, being the most relevant the likes, comments, shares, saves, reach, impressions, as well as interactions with the publications, which were considered for analysis of this work. Such metrics serve to analyze the performance of the profile and are available for monitoring the accounts reached with each publication and the interactions and information about the audience^(24)^.

Likes is a metric that indicates the network users who viewed the publication and liked the content, and Shares is a metric that verifies when someone has sent a Feed publication to another person or shared it to their own Story. Save is a feature for “saving” a viewed post on your Feed, so you can easily access it later. Comments are direct interactions with the publication, in which the user can give opinions and criticize the content. The sum of likes, comments, shares, and saves is called “interactions with the publications”^(15,24)^.

When it comes to reach and impressions, the metrics are quite similar. Impressions refer to the number of times a publication was viewed, without differentiating if it was seen more than once by the same user. Reach, however, does not calculate repetitions by the same user, measuring only the first time he or she had contact with the publication.

### Study protocol

The data captured through the metrics generated by Instagram itself were classified into: audience characteristics; themes and audience interaction with the publications; interactions with users; and interactions with the Reels tool.

There were 65 posts, whose subjects were classified into ten thematic categories to identify which one was the most liked, so it was not an object of this research to deepen analysis on the other categories. They were: 1. The covid-19 pandemic: understanding it for self-protection - which encompassed vaccination, information about the early treatment kit, and skin care; 2. The use and abuse of alcohol and other drugs; 3. Other Topics - diseases (sickle cell anemia, back pain, and fibromyalgia); 4. Mental health; 5. Sexuality, gender, and reproductive health; 6. STIs and AIDS; 7. LGBTQ+ health; 8. Black population health and racism; 9. Other themes - health promotion (food, sleep, and physical exercises); 10. Commemorative themes (SUS [Brazilian Unified Health System] Pride and World Health Day).

The Quiz is held one or two days prior to the topic of the week’s main publication. It contains statements for which followers can answer whether they are true or false, to analyze their knowledge on the subject.

The Reels function is used for quick and creative dissemination of the content presented to the platform’s users, since its reach extends to users who do not follow the “@*resenhadasaude*” profile. The themes already covered were about the second dose of the vaccine, myths about menstruation, and contraceptive methods.

### Analysis of results

Descriptive and percentage statistics were applied to analyze the data, displayed in graphs and tables.

## RESULTS

### Followers

**Figure 1:**
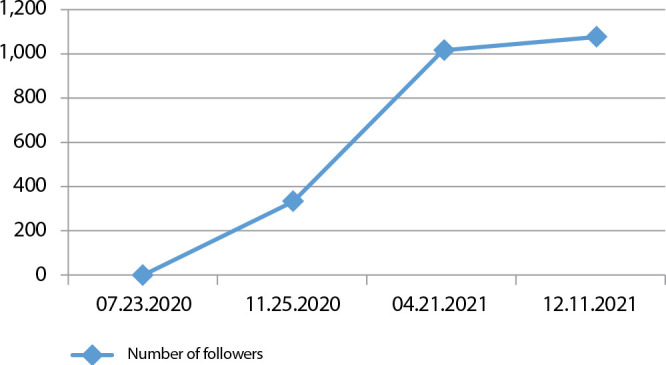
Number of followers in relation to dates

*Source: Instagram, 2021.*

The number of followers adds up to 1,175, located in several cities around the country, mainly in: Rio de Janeiro - RJ (44.1%), São Gonçalo - RJ (5.8%), Duque de Caxias - RJ (2.4%) and Niterói - RJ (2.4%). The growth curve is seen in Figure 1.

**Figure 2:**
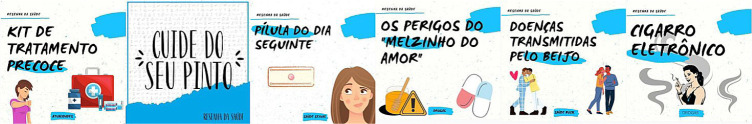
Publications’ covers with themes most liked by the public

*Translation of posts from the left: Early Treatment Kit; Take care of your dick; Morning after pill; The dangers of “Honey Supplements”; Diseases transmitted by kissing; Electronic cigarette.*

*Source: Instagram, 2021.*

It can be seen that from July 23 to November 25, 2020, approximately four months, the number of followers had not reached 400, whereas in the period from November 25, 2020, to December 11, 2021, the virtual tool exceeded one thousand followers. The rise from 332 on 11/25/2020 to 1,075 on 12/11/2021 represents a percentage increase of 223.79%. As of 4/21/2021, the followers were captured organically, that is, following was not done by young academics to publicize the page. Gender distribution of the followers is 70.90% female and 29.10% male.

The age range of the followers was important in defining the themes to be covered on the tool and shows a concentration among the public of teenagers and young people: 6.80%, between 13 and 17 years; 45.50%, aged between 18 and 24 years; 33.80%, between 25 and 34 years; 7.80%, between 35 and 44 years; and 6.10%, aged 45 years or more.

### Publications

The profile has 65 posts on various topics of interest to adolescents and young people, in addition to addressing commemorative dates. Those with the most likes - that is, those for which the public showed the most interest - were those related to the pandemic of covid-19, such as vaccination and treatment; and those related to sexual health and drugs.

Table 1 shows significant growth in impressions, likes, shares, and saves, which reveals the diversification in access and use of the Instagram profile “@resenhadasaude”; as to the potential of public interest for the profile. The metric referring to growth, marked with an asterisk (*), refers only to the period from 04/21/2021 to 12/11/2021, being the first date in the table, that is, the moment when data collection started.

**Table 1:** Comparison of data regarding the interactions of the public with the posts made on the Instagram profile “@*resenhadasaude*”, Rio de Janeiro, Rio de Janeiro, Brazil, 2021

Interaction	11/25/2020	04/21/2021	12/11/2021	Growth (%)
Likes	407	1,162	2,173	433.90
Comments	----	139	266	91.36^*^
Shares	196	444	680	246.94
Saves	52	96	191	267.31
Interactions with publications	----	1,841	3,310	79.79^*^
Reach	----	6,918	14,633	111.52^*^
Impressions	2.776	8,910	18,717	574.24

### Interaction with users and profile evaluation

Initially, interaction with users was limited to comments received on posts and private messages sent to the administrators’ message box. To expand interaction, the administrators created a form using the Google Forms tool so that followers could submit questions anonymously, avoiding any inhibition. However, so far there have been no completed forms submitted by the public.

The Quiz contains statements for the followers to answer whether they are true or false. The answers help managers to emphasize the correct information regarding the mistaken beliefs presented by the public. In Table 2, the correct answer is highlighted in gray.

**Table 2:** List of answers from the weekly Quizzes by theme and question, Rio de Janeiro, Rio de Janeiro, Brazil, 2021

Post	Affirmation	True (No. of answers)	False (No. of answers)
Condoms	The use of two condoms increases protection.	3	23
	The condom squeezes the penis.	3	23
	The condom can be used more than once.	1	27
	The condom is one of the best ways to prevent STIs.	23	3
Candidiasis	A poor diet contributes to the development of candidiasis.	34	6
	There is only vaginal candidiasis.	8	28
	Stress does not contribute to the development of candidiasis.	8	26
	Candidiasis is associated with lack of personal hygiene.	17	20
	Candidiasis is an STI.	18	18
Use of masks	Wearing the mask in open places is not important.	6	35
	A surgical mask can be used under the FFP2.	15	26
Do you know your body?	There is no difference between vaginal discharge and vaginal secretion.	12	22
Menstrual cycle	Menstrual cycle is the days that you menstruate.	6	29
	The menstrual cycle is generally 28 days long.	34	1
	During the fertile period, ovulation occurs.	30	3
	The fertile period does not occur every month.	4	28
Peace culture	Having empathy is an attitude that favors peace.	27	1
	Racism is not a type of violence.	3	26
	When we promote peace, we prevent violence.	27	2
	Violence should be treated as something normal.	3	26
Eating disorders	Eating disorders are nonsense.	2	49
	Eating disorders can cause nutritional problems.	43	4
	Anorexia and bulimia are the same thing.	4	47
Have you heard of the Billings Method?	All contraceptive methods prevent against sexually transmitted infections.	5	27
	The Billings Method does not depend on your self-knowledge.	13	17
	It is important to know your menstrual cycle.	30	1
	It is indifferent to be aware of vaginal secretions.	5	25
The harm of over-consumption of energy drinks	It's okay to mix energy drinks with alcoholic beverages.	6	26
	Energy drinks are good for your health.	8	27
	Energy drinks have a lot of caffeine and sugar.	31	3
What do you know about HPV?	HPV is a life-threatening disease.	33	4
	Using a condom protects against HPV infection.	33	5
	There is no vaccine against HPV.	9	30
	SUS offers a vaccine against HPV.	37	2
Syphilis	Syphilis is an STI.	35	1
	Syphilis has no cure.	13	24
	Syphilis treatment is very expensive.	7	30
	A pregnant woman with syphilis can pass the disease to her baby.	34	5
The morning-after pill	The morning-after pill is recommended only in cases of emergency.	22	1
	The morning-after pill can be used several times within the same month.	2	21
	Morning-after pills are abortifacient.	6	15
	The morning-after pill and the daily contraceptive are the same thing.	0	22
Intimate hygiene	You can use any soap for intimate hygiene.	5	37
	Douches can be used to sanitize the vagina.	8	31
	It is not necessary to dry the penis after urinating.	7	33
	The phimosis impairs the hygiene of the glans.	31	7
Covid-19 and its variants, what to do?	It is normal for a virus to suffer mutations.	33	3
	All variants are hazardous to health.	17	14
	Vaccination is not important in protecting against variants.	7	26
Electronic cigarette	Electronic cigarettes are not bad for your health.	2	24
	Some vapes include nicotine.	22	0
		2	21

*STI - Sexually Transmitted Infection; HPV - Human Papillomavirus.*

**Table 3:** Data relating to videos (Reels) from the “@*resenhadasaude*” Instagram profile, Rio de Janeiro, Rio de Janeiro, Brazil, 2021

Metric	Second dose of the vaccine	Myths about menstruation	Methods of contraception
Likes	266	97	136
Comments	21	9	3
Reproductions	9,070	1,787	5,658
Shares	44	4	12
Accounts reached	8,651	1,632	5,766

### Reels

Instagram’s video tool allows for a greater dissemination of content, expanding health promotion in a fun way, using music and trends.

## DISCUSSION

Access to the “@*resenhadasaude*” profile was 41.8% higher by the female audience. There is a lower demand from men for health care in the services intended for this purpose; and the reasons for this may be several, such as the opening hours of health units coinciding with the users’ work; gender stereotypes, which determine that the disease means fragility of the body and, thus, a self-perception of man as a more vulnerable person. In addition, there is the representation of masculinity as being less subject to health care^(25)^.

The cited research refers to the search for in-person care, but it is observed that the search for information on health care through virtual means was also greater by the female audience. Men’s lower search for health care information remained lower, despite considering that the reasons that keep them away from in-person care do not exist when it comes to the virtual environment, whose access is easier and anonymity can be maintained.

While distanced from in-person care, the possibility of welcoming this public on the Internet is heightened, promoting health and nursing care in this environment. An example is that, despite this difference in the demand for health services between genders, the percentage of men who accessed the content published on the page “@*resenhadasaude*” is relatively high. Perhaps this is due to the publications aimed at this gender, such as: “Take care of your dick”, classified in the thematic category “Sexuality, gender, and reproductive health”, whose purpose was to teach and warn about the importance of penile hygiene - one of the five most liked and engaged publications in the profile, published on October 29, 2020.

Social media needs to be enhanced to expand the dissemination of health information and promote men’s engagement. Research conducted on Twitter posts around the time of Movember, an annual event aimed at raising funds and awareness of men’s health issues, found that health content was rarely shared among individuals in Canada, the United States, and the United Kingdom, with other topics predominating in their conversations. The authors recommend investing in social networks to engage men in productive conversations about their health^(26)^.

As per age group, the one that most accesses the content ranges from 13 to 34 years, which represents a young-teen and young-adult audience. The profile’s target audience - teenagers and young people - composes majority among the followers, but it also attracts other age groups, which ratifies the information that social networks can reach a diverse audience and capillarize knowledge^(14,27)^. Access to the page “@*resenhadasaude*” proves to be a relevant tool to complement the knowledge about sex education of adolescents and young people, a theme not always addressed in the family and schools, due to ignorance or taboo.

As the tool became better known, the more the number of followers grew, as, within five months, the increase was quite representative. This indicates approval and expansion of the project, which reached a greater number of users and, consequently, a greater diversity of people. An example is the followers’ location, because, although most of them are in Rio de Janeiro (RJ), there are followers in other states in the Southeast Region, such as São Paulo (SP); in the North Region, such as Marabá (PA); and in the Northeast Region, such as Salvador (BA). Although the initial focus of the project was the city of Rio de Janeiro, it can be seen, therefore, that other states of the country were eventually reached. In this sense, Instagram can also serve to expand the role of nurses as disseminators of scientific knowledge.

The dissemination of scientific knowledge among the population is lacking, while the public’s interest in science and technology is identified, in direct relation to scientific education^(28)^. The good use of social media by nursing professionals and academics, guided by the ethical and legal limits that regulate the profession’s practices, can add social and professional recognition with the population, by the commitment to the dissemination of science and its application in health care.

The metrics provided by Instagram regarding audience interactions indicate a validation of the work by the audience, nurses, and academic nursing profile followers. The audience interacts, comments, and disseminates the content, becoming active agents in the learning process and scientific dissemination, while also collaborating with the expansion of knowledge conveyed by the page. Conversations and debates with adolescents and young people have been applied as a methodological possibility to provide dynamism to communication, revealing itself as a productive pedagogical tool in the physical classroom context in face-to-face teaching, and an interesting practice of approximation between subjects in pedagogical daily life^(29)^.

If we conceive Instagram as a democratic space for conversation and debate on various topics and, especially about health, it is understood that it is possible to have a displacement of the physical space of the classroom or waiting rooms of health services to the virtual space, where people can access content that help them reflect on their own and other people’s care and have subsidies for health promotion. Therefore, Instagram, in this case, under the management of technically prepared, responsible, and committed groups, can democratize the debates about health and care and provide access to safe and quality information. The significant growth in followers of the “@*resenhadasaude*” profile page - in which some metrics more than doubled their value, and the metric on content impression more than tripled its previous value - shows not only the public’s interest in the themes, but their trust towards the page.

Research conducted in 2013 on the use of social networks at school, with 50 teachers from 46 public and private schools in 17 municipalities in the state of Rio de Janeiro, found that most schools (48%) still did not allow the use of social networks, either by teachers or students. In some schools, its use was restricted only for students (32%); and in only 20%, it was allowed for both teachers and students^(30)^. Seven years have passed since this research, and it is worth pointing out the undeniable function of social networks as a complement in the educational process. They also served in granting access to information on health promotion and disease prevention, whether due to misor incomplete information, especially when there was limited in-person access to educational institutions and to the health units themselves, due to the covid-19 pandemic.

Furthermore, it is important to consider that the serious health crisis that the world has been going through since the end of 2019 has shown various social fragilities and vulnerabilities. In this sense, social media have gained a place of importance by permitting both the approximation of people who were required to maintain physical distance and the educational process, with readaptations for the remote modality, despite it not being ideal, egalitarian, and democratic^(31)^. Nevertheless, it is necessary to explore the media for what they are useful for, and to manage them in favor of society and the common good through a good use of the available tools.

Tackling this debate, a bibliometric and scientometric research aimed to identify the temporal and thematic behavior of media studies in information science. This initiative arose due to the transformations observed in the media field, driven by ICTs. This occurred based on the understanding that technical and language procedures enabled the growth of the flow of information, its visibility, and circulation in society. The author adds that the occupation of the field of media in contemporary societies should be understood as strategic, although further studies are needed to expand this knowledge^(32)^.

The fact is that access to social media by a diversified public seems irreversible, since, considering the data from this research, in a few months there was a significant increase in the search for information through the tool “@*resenhadasaude*”. The concern should be based on the credibility of the content disseminated in this and other media. Hence, the need and care to verify the authorships of the publications, their institutional and professional links.

The transformations in the media field, driven by the ICTs and resulting from technical and language procedures, have increased the flow of information, as well as its visibility and circulation in society. Fundamentally, the occupation of the media field in contemporary societies has become strategic. Nevertheless, the relationship between the field of information science and this theme remains little known.

With the increase of communicability with the public, doubts could be easily clared, enriching the contact with the management team of the profile “@*resenhadasaude*”. The development of Quizzes with weekly themes allowed the detection of doubts and misunderstandings in the knowledge of some of the followers, since, in the first Quiz held, there were followers imbued with the belief that the male condom squeezes the penis, showing a deficit in knowledge about the sexual health theme. The diagnosis of knowledge deficit allows health educators to promote and increase the agendas on the topics that generate most questions and, thus, feed the publications and enhance the dissemination of technical and scientific information to the population.

Results of a research in oral health also showed that the Instagram social network is an efficient means of communication, being both an alternative to disseminate health information and a tool for health surveillance^(33)^. It is easily accessible, with a wide audience, and can be used to disseminate quality and reliable information^(27)^. In this sense, a successful educational campaign strategy with the use of Instagram was observed in Iran during the covid-19 pandemic, revealing the important role of a good use of social networks in the dissemination of mass information^(34)^. This shows that, associated with other media and platforms, this social network contributes to expand communication and dissemination of information in the field of public health to a wider audience^(14)^.

However, even if the educational and inclusive potential of scientific dissemination and its strategic importance is identified, it is recognized as timely and necessary that there be a greater encouragement of strategies that make it feasible, through face-to-face and remote means, with public policies specific to this area^(35)^. Furthermore, regarding the Internet’s social media, it is recommended that there be a digital inclusion policy capable of ensuring that all social strata have access to the Internet, expanding the opportunities for communication and knowledge in an equitable and egalitarian way, as a truly democratic society requires.

A study on the use of social networks in the nursing work process shows that one should identify the best practices and learn to use them to make better use of them, and that such networks can provide a more accessible and useful method in the dissemination of health education content^(36)^.

Instagram presents itself as a technology to be used for educational purposes, addressing well what is required in the field of information and scientific dissemination^(12-13,20)^. Added to other educational strategies widely applied by nursing, the use of this network for educational purposes and dissemination of information can express a new field of practice for nursing professionals and students, autonomously, in health promotion through social networks. The public can benefit from the democratization of access to health since most Brazilians have access to Instagram. It is crucial that the profiles be well identified in terms of their administrators and their affiliations, to create relationships of trust with the public and to contribute to strengthening the social image of the profession.

### Study limitations

This research brings to light data from a particular health education profile on the Instagram social network, aimed at adolescents and youth, so the analysis and conclusions are restricted to this experience. The expansion of the debate requires that other profiles serve as a research field to know the access and acceptability of other themes and types of audiences. Also, the social network does not allow access to complete data on location and age groups, allowing visibility of only those considered the main ones.

### Contributions and Implications for Nursing

The contribution of this research lies in the field of health education and the promotion of care, in which the nursing role is traditional and effective. It shows the opening of a field of work for nurses and nursing students to broaden the scope of their actions, making it possible to reach a larger audience and greater social penetrability, with increased dissemination of their work on behalf of the health of the population.

## CONCLUSIONS

The audience that accesses the project’s Instagram are teenagers, youth, and young adults, with a predominance of the female audience. Mostly, the project reaches people from the Southeast Region, but there are followers from the Northeast and North Regions of the country. To increase the reach of the information, it is necessary to focus on the dissemination to the public in the states where there are already followers, but mainly in the regions where the profile is not yet known, that is, the South and Midwest. For this, the tools and resources of Instagram itself can be used to provide this impulse. The metrics made available by Instagram point to the validation of the work done by the project in the field of health education, in terms of public interest. This social network is a technology to be also used for educational and information dissemination purposes and expresses a new field of nursing practice, of an autonomous nature, in health promotion through social networks. Nevertheless, in addition to showing that Instagram can be used to disseminate information and promote health education, it is necessary that the manager of the page monitors its metrics to evaluate the reach of the profile and boosts it seeking a wider audience.
